# A Spatial Study of Head and Neck Cancer Incidence Rate in Fars Province (the South of Iran): 2007-2020 

**DOI:** 10.30476/dentjods.2025.103364.2444

**Published:** 2025-12-01

**Authors:** Sara Maroufi, Fahimeh Rezazadeh, Sara Haghighat, Naeimehossadat Asmarian, Alireza Kheiri, Hila Raeisi, Mohammad Afifian, Ardalan Banani, Setareh Valanik

**Affiliations:** 1 Postgraduate Student, Dept. of Oral and Maxillofacial Medicine, School of Dentistry, Shiraz University of Medical Sciences, Shiraz, Iran.; 2 Oral & Dental Disease Research Center, Dept. of Oral and Maxillofacial Medicine, School of Dentistry, Shiraz University of Medical Sciences, Shiraz, Iran.; 3 Student Research Committee, School of Dentistry, Shiraz University of Medical Sciences, Shiraz, Iran.; 4 Anesthesiology and Critical Care Research Center, Shiraz University of Medical Sciences, Shiraz, Iran.

**Keywords:** Spatio-temporal analysis, Head and neck cancers, Incidence, Epidemiology, Squamous cell carcinoma, Iran

## Abstract

**Background:**

Head and neck cancers (HNCs) are among the top ten most common cancers globally. There is a clear geographical bias in the prevalence of HNCs. More than two-thirds of HNC cases worldwide occur in low- and middle-income countries. Due to the high prevalence of HNCs in Fars province.

**Purpose:**

The purpose of this study was to assess the spatial pattern of HNC incidence rates by modeling both the effects of spatial dependence between neighboring regions and risk factors in a Bayesian Poisson model (BYM).

**Materials and Method:**

In this cross sectional study, from the Center of Cancer Registry in Fars province, data was collected from 1,821 patients diagnosed with HNC. The effects of spatial structure were modeled in a Bayesian spatiotemporal hierarchical model to determine the relative risk and trend of HNC incidence rates. The maps were created geographical variations of HNCs incidence across the 29 counties of the province with classical Standardized Incidence Rate (SIR), BYM model, and spatiotemporal model.

**Results:**

The highest crude incidence rates were 0.55 and 0.16 cases per 1,000-person population for HNC and squamous cell carcinoma (SCC), respectively. Spatially, the highest relative risks for HNC and SCC were estimated at 1.36 and 1.34, respectively, in the county of Shiraz, the capital of Fars province. The lowest relative risks for HNC and SCC were estimated at 0.39 and 0.46 per 1,000 persons, respectively, in Gerash County in southern Fars. The findings showed an increasing trend in the HNC incidence rate and a decreasing trend in SCC incidence in this province.

**Conclusion:**

Spatial analysis of HNCs revealed a high incidence rate in the northern and northeastern parts of Fars province, which may be attributed to the effects of lifestyle factors and certain pollutants in the region's cold air.

## Introduction

Cancer is the second leading cause of death worldwide. Head and neck cancers (HNCs) are among the top ten most common cancers globally, responsible for more than 650,000 cases and 350,000 deaths annually [ 1 ]. HNCs are a heterogeneous group of malignancies with varying tumor biology, prognosis, and therapeutic responses [ 2 ]. These cancers may affect different parts of the head and neck, including the oral and nasal cavities, lips, pharynx, larynx, paranasal sinuses, thyroid, and salivary glands. The most common sites for HNCs and oral cancers are the pharynx and tongue, respectively [ 3 - 5 ].

There is a clear geographical bias in the prevalence of HNCs. More than two-thirds of HNC cases worldwide occur in low- and middle-income countries [ 3 , 6 ]. The female-to-male ratio of HNC prevalence varies across studies, but most suggest higher rates in men. However, in certain malignancies, such as thyroid cancer, women have a higher incidence . The most common age group affected by HNC is those in their 5th and 6th decades of life [ 11 - 12 ].

Several risk factors are implicated in HNCs, including the use of tobacco and alcohol, and infections with viruses such as Epstein-Barr virus (EBV) and human papillomavirus (HPV). Other risk factors for HNCs include radiation and occupational exposures, socioeconomic status, periodontal disease, vitamin deficiencies, and dietary habits [ 13 - 14 ].

Various studies have analyzed the prevalence and incidence of HNC or oral cancer using cross-sectional methods or by reporting only descriptive data specific to the most common cancer, squamous cell carcinoma (SCC), or focusing on a single institution or center . Such analyses have been applied to various cancers, including skin cancer [ 17 ], colorectal cancer [ 18 ], and breast cancer [ 19 ]. However, to the best of our knowledge, no spatial study has yet used Bayesian spatial analysis for HNCs in Iran. Therefore, in the present study, we estimated the incidence rate of HNC in Fars province, located in southern Iran, and investigated the trend of HNC incidence over 14 years (2007–2020) using Bayesian spatial analysis and the Besag, York, and Mollie (BYM) model [ 20 - 21 ]. 

## Materials and Method

**Figure 1:**
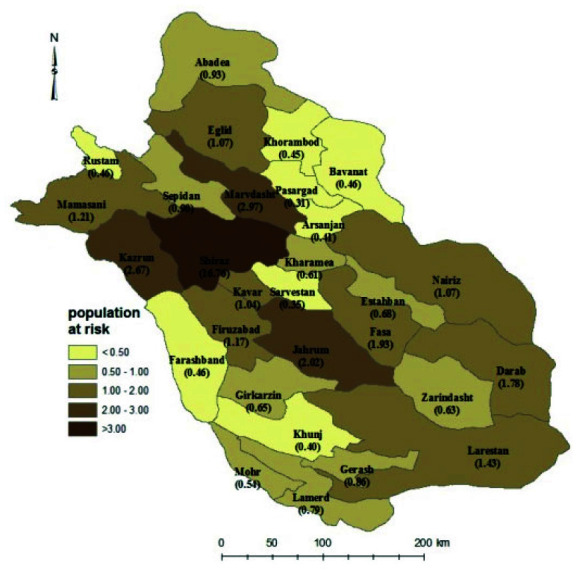
Geographical location of 29 counties in Fars province and the population at risk (1/100,000)

We collected data on the incidence of new cancer cases over 14 years, from 2007 to 2020. Data were obtained from the Fars Center for Cancer Registry. Fars province is located in the southwest region of Iran and is subdivided into 29 counties (Figure 1). The data were gathered from all pathology laboratories in Fars province and included demographic and clinical details for each patient with a confirmed cancer diagnosis. Each type of cancer is identified by a code according to the ICD-10 coding criteria. Among all types of recorded cancers, those that define HNCs were included in this study (Ethical Code: IR.SUMS.REC.1393. 7582). 

### Statistical Analysis

**Figure 2:**
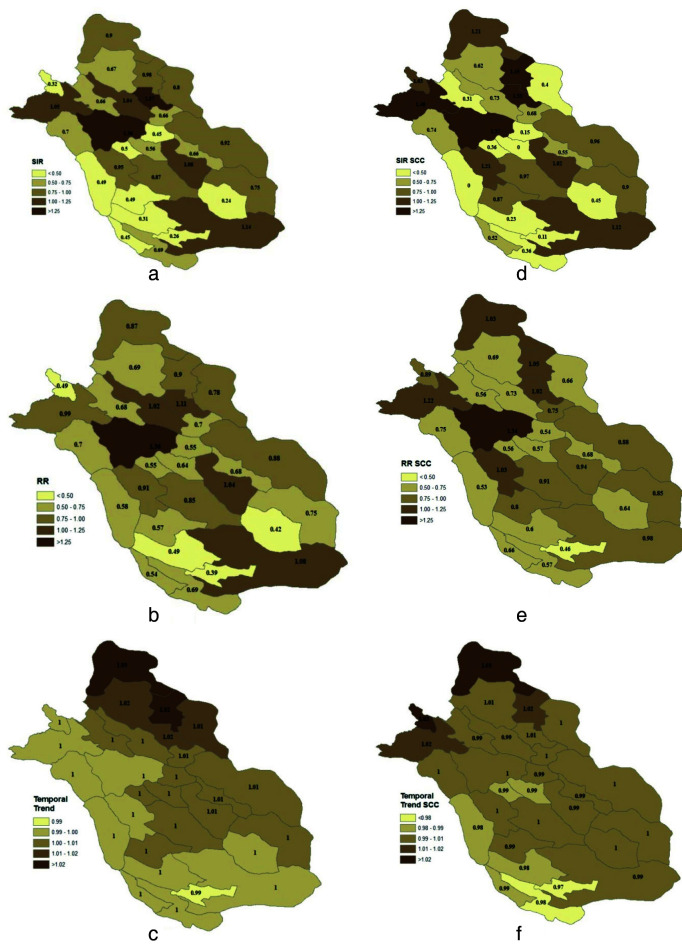
**a:** Head and neck cancer (HNC) incidence rate across the 29 counties of Fars province with classic standardized incidence rate (SIR), **d:** Squamous cell carcinoma (SCC) incidence rate across the 29 counties of Fars province with classic SIR, **b:** relative risk by BYM model, of HNC incidence rate and **e:** SCC incidence rate, **c:** Posterior temporal trend of HNC incidence rate, and **f:** posterior temporal trend of SCC incidence rate

In this study, the geographical variations in HNC incidence rates across the 29 counties were analyzed. The maps were created geographical variations of HNCs incidence across the 29 counties of the province with classical Standardized Incidence Rate (SIR) (Figure 2a, d), BYM model (Figure 2b, e), and spatiotemporal model (Figure 2c, f). The maps should be interpreted considering that different shades are proportional to the incidence rate value. In other words, the darker the area shows the higher incidence of HNCs. The Standardized Incidence Rate (SIR) was calculated for each county using the direct method. Since the time coefficient is in the logarithm of the odds ratio, we used the Exponential function to transform it into odds ratios. The observed number of new HNC cases in each geographic unit (county) was used for this analysis. Statistical analyses were performed using SPSS version 19.0 software. 

## Results

We identified 1,821 new cases of HNC among residents of Fars province between 2007 and 2020. Of these, 57.1% were male. A total of 924 patients (51%) were from Shiraz city, the capital of Fars province. Table 1 presents the number of new HNC cases, crude rates per 1,000 persons, the results of the BYM model, and spatio-temporal regression analysis for different counties in Fars province.

**Table 1:** Model estimates of oral incidence in Fars, south of Iran from 2007 to 2020; SCC: squamous cell carcinoma

County	Number of oral cases	Number of SCC cases	Population	Crude incidence rate per 1,000 pop	Crude incidence rate per 1,000 SCC	Relative Risk general	Relative Risk SCC	δ general	δ SCC
Abadeh	34	12	92959	0.37	0.13	0.87	1.03	0.02	0.03
Arsanjan	11	3	41488	0.27	0.07	0.70	0.75	0.01	0.00
Estahban	18	4	67875	0.27	0.06	0.68	0.68	0.00	-0.01
Eqlid	29	7	106664	0.27	0.07	0.69	0.69	0.01	0.01
Bavanat	15	2	46434	0.32	0.04	0.78	0.66	0.01	0.00
Jahrum	71	21	202445	0.35	0.10	0.85	0.91	0.00	0.00
Khorambod	18	7	45381	0.40	0.15	0.90	1.05	0.02	0.02
Khunj	5	1	40296	0.12	0.02	0.49	0.60	-0.01	-0.02
Darab	54	17	177938	0.30	0.10	0.75	0.85	0.00	0.00
Rustam	6	5	45686	0.13	0.11	0.49	0.89	-0.01	0.03
Zarindasht	6	3	62817	0.10	0.05	0.42	0.64	0.00	0.00
Sarvestan	8	0	35313	0.23	0.00	0.64	0.57	0.00	-0.01
Sepidan	24	3	90339	0.27	0.03	0.68	0.56	0.00	0.00
Shiraz	924	244	1675873	0.55	0.15	1.36	1.34	-0.01	0.00
Farashband	9	0	45678	0.20	0.00	0.58	0.53	-0.01	-0.01
Fasa	84	21	192946	0.44	0.11	1.04	0.94	0.00	0.00
Firuzabad	45	15	116622	0.39	0.13	0.91	1.03	0.00	0.00
Qir and Karzin	13	6	65148	0.20	0.09	0.57	0.80	0.00	0.00
Kazrun	75	21	266564	0.28	0.08	0.70	0.75	0.00	0.00
Larestan	66	17	142788	0.46	0.12	1.08	0.98	0.00	-0.01
Lamerd	22	3	78692	0.28	0.04	0.69	0.57	-0.01	-0.02
Marvdasht	125	23	297399	0.42	0.08	1.02	0.73	0.00	0.00
Mamasani	51	19	120622	0.42	0.16	0.99	1.22	0.00	0.02
Mohr	10	3	54413	0.18	0.06	0.54	0.66	-0.01	-0.01
Neyriz	40	11	107406	0.37	0.10	0.88	0.88	0.01	0.00
Pasargad	17	5	30632	0.55	0.16	1.11	1.02	0.01	0.01
Gerash	9	1	86460	0.10	0.01	0.39	0.46	-0.01	-0.03
Khavar	21	4	104000	0.20	0.04	0.55	0.56	0.00	-0.01
Kharameh	11	1	61000	0.18	0.02	0.55	0.54	0.00	-0.01

HNCs were most commonly found in the lymph nodes and oral cavity, comprising 41.9% and 24.6% of all cases, respectively. The most frequent types of HNC in our study were lymphomas and carcinomas, accounting for 45.6% and 42.4% of all cases, respectively. Melanoma and leukemia were the least common types, each making up 0.3% of all cases. SCC was the most common histologic subtype, representing 25.7% of all cases. Hodgkin and non-Hodgkin lymphoma were the second and third most common cancer subtypes, comprising 22.8% and 15.3% of all cases, respectively. The most common age group across all HNC cases was 50-60 years, which accounted for 17.5% of all cases. In SCC cases, the most common age group was 70-80 years, comprising 23.4% of SCC cases.

The average SIR and relative risks for HNCs were 0.73 and 0.75, respectively. Based on Figures 2B and 2E, Shiraz is at higher risk than other counties. This city had the highest SIR and relative risk for all HNCs, with values of 1.36 and 1.36 (standard deviation= 0.04), and for SCC, the values were 1.37 and 1.34 (standard deviation= 0.09), respectively. Gerash County, in the southeast of Fars province, had the lowest relative risk, with values of 0.39 for general HNCs (standard deviation= 0.09) and 0.46 for SCC (standard deviation= 0.17). Figures 2C and 2F show the temporal trend of the incidence rate across different regions of Fars for the period 2007-2020. The estimated time coefficients in this model were 0.004 (general) and 0.003 (SCC).

The odds ratios corresponding to the time coefficient are 1.004 (general) and 0.997 (SCC) for each successive year. These findings suggest that while there is an increasing trend in the incidence rate of general HNCs and a decreasing trend for SCC in Fars, these trends are relatively uniform.

Another important observation from Figures 2c and 2f is the steady increase in the incidence of relative risk over time in the northern part of the province. A key distinction between Figures 2b and 2e, and Figures 2c and 2f, is the interactive relationship between time and incidence rates in different geographic regions. Figures 2b and 2e show an average over the 14 years, resulting in higher overall incidence rates in the central regions of Fars. In contrast, Figures 2c and 2f reflect relative changes over time, showing a geographical shift in the incidence rates. 

## Discussion

This study is the first to investigate the spatial pattern of HNC on a large scale over a 14-year period in the south of Iran. We analyzed the distribution of HNC cases in Fars province using the BYM model and recorded the relative risk of cancer incidence in each geographic region. We also reported descriptive statistics regarding age, gender, and the different types and subtypes of cancers. To the best of our knowledge, only one study has spatially analyzed an HNC type in Iran. Safavi-Naini et al. [ 3 ] examined the geographical distribution of nasopharyngeal cancer in Iran over 6 years, reporting that Gilan and Ilam provinces had the highest and lowest incidence rates of nasopharyngeal cancer, respectively. Their study did not include data on Fars province or other types of HNCs.

According to our results, 57% of all HNC cases were male, which is consistent with previous studies. Mirzaei et al. [ 4 ] investigated HNCs in Iran from 2003 to 2009 and found that all cancers had a higher incidence rate in men, except for thyroid cancer, which was more common in women. Similarly, studies from India and Canada also reported significantly higher incidence rates of HNCs in men [ 22 - 23 ].

In our study, the most affected age group was 50- to 60-year-old individuals. This pattern was consistent with findings in some previous studies. In the studies conducted by Larizadeh et al. [ 24 ] and Basirat et al. [ 25 ] in the northern and central regions of Iran, the most affected age group was the 6th decade of life. Interestingly, 26% of our cases were under 31 years old, a higher proportion than reported in other global studies. For example, in studies by Brandizzi et al. [ 26 ] and Bhattacharjee et al. [ 27 ], which examined HNC prevalence in India and Argentina, less than 4% of patients were under 30 years old. This higher rate in younger individuals may be attributed to the relatively high number of lymphoma cases among our study participants. Furthermore, a recent study by Shiboski et al. [ 28 ] in the United States reported an increase in the incidence of tongue and pharyngeal carcinomas in the 20-44 age groups over time.

Among all head and neck sites, the most common cancer site in our study was the lymph nodes (41.9%), due to the predominance of lymphoma. The oral cavity was the second most common site, accounting for 24.6% of cases. The tongue, nasopharynx, and salivary glands were the most frequently involved intraoral sites. Several studies in Iran have found that the tongue is the most commonly affected site in oral cancer [ 12 ]. In studies by Andisheh-Tadbir et al. [ 13 ], Falaki et al. [ 14 ], and Sargeran et al. [ 15 ], the tongue accounted for 53%, 66%, and 50% of all oral cancers, respectively. However, other studies reported different patterns. In Razavi et al.'s study [ 29 ] in Isfahan, Iran, the tongue accounted for only 18% of cases, which is quite different from our findings. Similarly, in Delagranda et al.'s study [ 30 ] on Reunion Island (France), cancers of the salivary glands and tongue base had lower incidence rates than other sites. These differences may be attributed to variations in study design and the specific cancer types assessed (HNCs vs. only oral cancers).

Lymphomas and carcinomas accounted for 45.6% and 42.6% of all HNC cases in our study, respectively, making up 88% of the total. Sarcomas were rare, comprising less than 6% of cases, while leukemia and melanoma were the least common types, each accounting for only 0.3%. The high rate of lymphoma in our study is notable, as other studies did not assess lymphoma as part of HNC. In Adoga et al.'s study [ 31 ] in Nigeria, 92.6% of cases were carcinomas, and only 5.7% were lymphoma. Similarly, Gupta et al. [ 32 ] found that only 8.25% of all cancers were lymphoma. A study in northern Iran also reported an increasing rate of lymphoma malignancy in the head and neck region over time, which aligns with our findings [ 25 ].

The most common histologic type of cancer in our study was SCC, with 485 cases, predominantly in men (54.6%). The mean age of SCC patients was 61.57± 16.43, with 40% of lesions located on the tongue. These findings are consistent with previous studies. In Andisheh-Tadbir et al.'s study [ 13 ] in Shiraz in 2008, the mean age of SCC patients was 56.9±15.5, and most were male (1.4:1). In Bakhtiari et al.'s study [ 33 ] in Ahvaz, southwest Iran, 74.1% of SCC patients were male, and the mean age was 63.19±16.33. The most common lesion site was the larynx (37.9%), and the tongue accounted for only 8.1%, differing from our results. Hodgkin and non-Hodgkin lymphoma were the second and third most common histological types of HNC in our study, consistent with findings in other studies. For example, in Kerman province, SCC was the most frequent histologic type of HNC, with lymphoma being the second most common malignancy [ 6 ].

Using the BYM method, we calculated the SIR for each county in Fars province. The overall SIR for HNC in Fars was 0.73, and the relative risk was 0.75 (ranging from 0.39 to 1.36). Spatial analysis revealed that Shiraz had the highest risk for HNC (1.36) and SCC (1.34). Shiraz, being the capital and largest city of Fars province, is more likely to experience higher rates of cancer due to factors such as environmental pollution, unhealthy lifestyles, and stress. Additionally, the availability of advanced diagnostic facilities may contribute to the higher number of cancer diagnoses in Shiraz. Gerash, located in the southeast of Fars, had the lowest relative risk for HNCs, which could be due to better nutritional habits and lower rates of smoking and alcohol consumption.

Over time, the incidence of oral cancer has been slowly increasing, but the rate of change differs by region. While Shiraz has experienced a relatively stable number of HNC cases, northern counties have seen an increase in recorded cases. This rising incidence in the northern counties may be linked to lifestyle or dietary changes in these areas. A recent review published in 2017 indicated that the overall incidence of oral SCC is increasing globally, with a greater rise in patients under 45 years old compared to older populations [ 34 ]. This suggests that clinicians should focus on diagnosing oral SCC in younger individuals. A study by Simard et al. [ 35 ] also reported a decrease in SCC incidence in 6 countries, including India and Thailand, while the rate remained stable in several South American countries.

One limitation of our study was the incomplete and inaccurate data in some patient records. Some records lacked important details, such as the patient's city of residence, and information about lifestyle habits and health status was often missing. As a result, we were unable to precisely determine risk factors for HNC incidence. Additionally, during the study period, Fars province underwent administrative changes, with some counties being split or merged, potentially affecting the accuracy of the data. Further research is needed to explore the factors contributing to the relatively high rate of cancer in the northern areas of Fars province. 

## Conclusion

Spatial analysis of HNCs revealed a high incidence rate in the northern and northeastern regions of Fars province. The most common types of HNCs in our study were lymphomas and SCC, with melanoma and leukemia being the least common. The most frequently affected age group was 50-60 years old. Shiraz, the capital of Fars, had the highest relative risk and SIR for HNCs. In contrast, Gerash County, located in the southeast of the province, had the lowest relative risk. Our findings indicated that although there were an overall increase in HNC incidence, with a rising trend in general HNCs and a slight decline in SCC incidence, the trends were relatively uniform. Notably, the relative risk of HNCs has been steadily increasing over time in the northern part of the province, while the central regions of Fars experienced higher overall incidence rates across the 14 years. The spatial shift observed over time suggests a changing geographical distribution of HNC cases.
